# *Dirofilaria repens* in the Spermatic Cord of a 5-Year-Old Boy: A Rare Pediatric Case in Italy

**DOI:** 10.3390/tropicalmed10070184

**Published:** 2025-06-29

**Authors:** Alessandro Franzò, Andrea Marino, Benedetto Maurizio Celesia, Roberto Bruno, Pieralba Catalano, Sebastiano Cacciaguerra, Stefano Reale, Bruno Santi Cacopardo, Giuseppe Nunnari

**Affiliations:** 1 Unit of Infectious Diseases, Department of Experimental and Clinical Medicine, ARNAS Garibaldi Hospital, University of Catania, 95122 Catania, Italy; alefranzosr@gmail.com (A.F.); bmcelesia@gmail.com (B.M.C.); roberto.bruno@unict.it (R.B.); cacopardobruno@inwind.it (B.S.C.); giuseppe.nunnari1@unict.it (G.N.); 2 Department of Pediatric Surgery, ARNAS Garibaldi Hospital, 95122 Catania, Italy; pieralba.catalano@gmail.com (P.C.); scacciaguerra@arnasgaribaldi.it (S.C.); 3 Istituto Zooprofilattico Sperimentale della Sicilia, 90129 Palermo, Italy; stefano.reale@izssicilia.it

**Keywords:** dirofilariosis, pediatric helminthiasis, vector-borne diseases, zoonotic diseases, pediatric zoonotic diseases

## Abstract

We report the case of a 5-year-old boy from a Sri Lankan migrant family in Catania, Italy, diagnosed with a *Dirofilaria repens* infection in the spermatic cord. The child presented with pain and swelling in the left inguinal area. Initial evaluation suggested orchiepididymitis, which was treated unsuccessfully with amoxicillin/clavulanate and NSAIDs. As symptoms worsened, torsion of the Morgagni hydatid was considered. An exploratory surgery revealed a firm mass in the left spermatic cord. Histopathological examination of the excised lesion showed fragments of a helminth within a granulomatous inflammatory reaction. Subsequent PCR analysis detected *D. repens* DNA. The patient fully recovered after surgical excision of the mass. Given the increasing incidence of human dirofilariasis, *D. repens* should be considered in the differential diagnosis of unexplained subcutaneous or inguinal nodules, especially in patients with a relevant travel history. This case highlights the importance of accurate diagnosis to avoid unnecessary invasive procedures or prolonged antimicrobial therapies. It represents one of the youngest pediatric cases with genital involvement reported in Italy, a country that accounts for half of the cases in Europe.

## 1. Introduction

*Dirofilaria repens* is a filarial nematode that primarily infects canids as definitive hosts, with humans serving as accidental hosts [1].

The parasite’s life cycle involves transmission through the bite of infected mosquitoes of the *Aedes*, *Culex*, and *Anopheles* genera, which act as vectors. In the mosquito, microfilariae develop into infective L3 larvae. When transmitted to a host, these larvae migrate to subcutaneous tissues to mature. In humans, most larvae do not survive to produce microfilariae due to the host’s immune response; however, they can form nodules at various sites [1].

While most human *D. repens* infections present in subcutaneous tissue, rare cases have been reported in the eye [2] and even more infrequently in the genitalia [3]. The parasite is endemic in Asia, Africa, and Southern and Eastern Europe, particularly in warmer, mosquito-endemic areas [1,4]. Notably, Italy accounts for approximately half of all human dirofilariasis cases reported in Europe [5].

Despite its growing prevalence, awareness of this zoonosis remains low, and it is often overlooked in the differential diagnosis of nodular lesions [6]. Genital involvement is exceedingly rare, particularly in children [7]. Here, we present the case of a 5-year-old boy with *D. repens* infection in the left spermatic cord, representing the youngest patient with this localization reported in Italy. This case underscores the need to consider parasitic infections when evaluating inguinal masses, even in young children, to ensure accurate diagnosis and appropriate management.

## 2. Case Presentation

**Figure 1:**
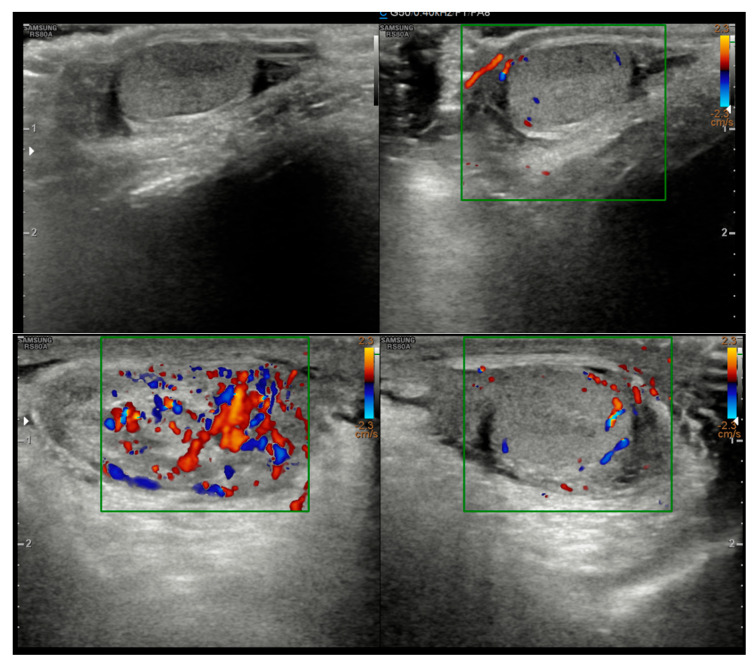
Initial ultrasound showing a thickened left spermatic cord with increased vascular signals.

**Figure 2:**
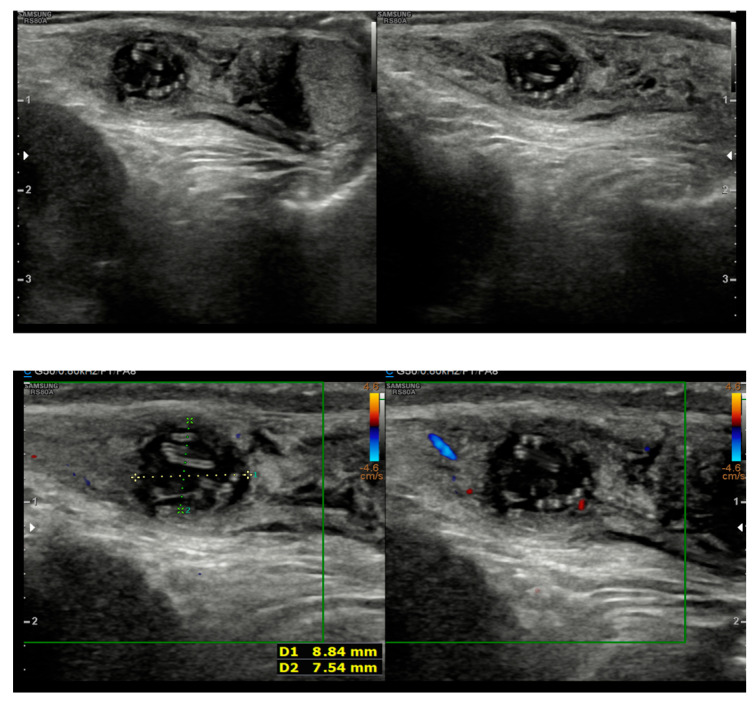
Follow-up ultrasound demonstrating a hypoechoic nodule containing linear hyperechogenic lines.

A 5-year-old boy, born in Catania, Sicily, Italy, to a Sri Lankan migrant family, was evaluated for left testicular pain and swelling. He had no prior hospitalizations or significant medical history. He was fully vaccinated. The symptoms were initially attributed to orchiepididymitis, as the left testis was edematous, painful, and hyperemic. The patient was treated with oral amoxicillin/clavulanate and nonsteroidal anti-inflammatory drugs (NSAIDs) for 10 days. There was mild improvement in swelling, but testicular pain persisted. Laboratory tests, including blood counts and inflammatory markers, showed only mild thrombocytosis (415,000/mmc). An ultrasound performed at the Pediatric Emergency Department of Garibaldi Nesima Hospital in Catania showed thickening of the left spermatic cord with increased blood flow on Doppler, while the testes appeared normal in morphology, and no hydrocele was present (Figure 1). Two weeks later, at a follow-up visit, the boy’s symptoms had worsened. A firm, elastic, mobile mass was palpated in the left inguinal/scrotal area, distinct from the right testis. The testis ultrasound at that time revealed a well-defined, round hypoechoic mass (measuring 8.5 mm) with linear hyperechoic structures within it (Figure 2).

**Figure 3:**
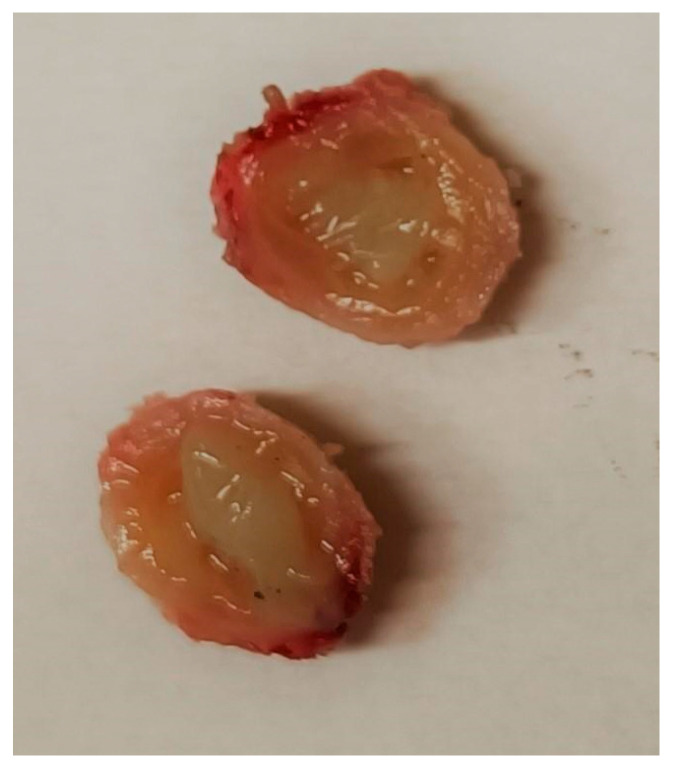
Surgical sample of the excised nodule in the left spermatic cord.

**Figure 4:**
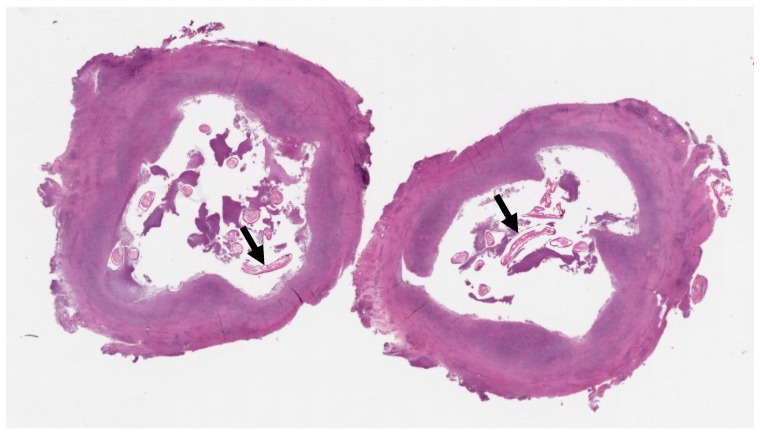
Histological section of a whitish centimetric nodule: Pseudocyst with chronic granulomatous inflammation; in the lumen, fragments of helminth (black arrows) and fibrino-purulent exudate.

The differential diagnosis at this stage included torsion or detachment of the appendix testis (hydatid of Morgagni) versus a granulomatous infection or neoplasm. Given the uncertainty and persistence of the mass, an exploratory surgical intervention was performed. The pediatric surgeons excised an encapsulated, tough mass from the left spermatic cord (Figure 3). The mass was not adherent to the testis or epididymis. Histopathological analysis of the nodule revealed a pseudocyst with chronic granulomatous inflammation, containing fragments of helminth, surrounded by fibrinous and purulent exudate (Figure 4).

As a result of our infectious diseases consultation and given the unusual finding and the young age of the patient, the specimen was sent for molecular analysis to the regional reference laboratory (Istituto Zooprofilattico of Palermo), where Polymerase Chain Reaction (PCR) detected *D. repens* DNA. Total DNA extraction was carried out from the sample tissue using a silica column kit [QIAamp DNA Mini Kit (QIAGEN, Hilden, Germany)]. The extracted DNA was employed for the amplification with four specific forward primers and a common reverse primer (Latrofa et al., 2012 and Passavia et al., 2018 [8,9]). Multiplex PCR cytochrome c oxidase subunit I (coxI) amplicons from the DNA extracted from the embedded tissue resulted in a band consistent with *D. repens* (479 bp). The amplified bands were analyzed by Sanger sequencing employing an ABI Prism 3500 capillary electrophoresis apparatus (Applied Biosystems, Foster City, CA, USA). The collected data was then analyzed by Chromas software v2.6.6 (Technelysium Pty Ltd., South Brisbane, Australia), considering 97% identity as the stringent parameter to identify the strains by data comparison with reference sequences. The BLAST v2.16.0 (Bethesda, MD, USA) analysis of the coxI sequences revealed a 99 to 100% identity compared to the sequences available in GenBank™. The sequencing experiment was repeated to confirm the sensitivity and specificity of the amplified bands (Supplementary Table S1).

Postoperatively, the patient recovered well. His pain resolved completely after the surgery, and no complications such as infections or recurrence of swelling were observed. At follow-up examinations over the subsequent months, the child remained symptom-free with no new lesions. We also conducted an ophthalmologic evaluation at follow-up to screen for any occult *Dirofilaria* lesions in the eye, which was normal. The family was advised on preventive measures, including mosquito bite avoidance and prompt evaluation of any new nodules.

## 3. Discussion

Human dirofilariasis is an emerging vector-borne zoonosis of increasing public health importance in Europe. The parasites *Dirofilaria repens* and *D. immitis* are filarial nematodes transmitted by mosquitoes, with canids serving as their natural definitive hosts [1]. While *D. immitis* is known for causing pulmonary disease in humans that can mimic lung tumors [10], *D. repens* typically causes benign subcutaneous nodules that develop over weeks to months [2]. These lesions can appear anywhere on the body but are most common on exposed areas like the face, neck, and limbs [1,4]. This case is significant as it involves the spermatic cord, a particularly rare localization for *D. repens* infection [7].

Our patient’s case adds to the very small number of pediatric dirofilariasis cases affecting the male genitalia. Between 1990 and 1999, sixty cases of human *D. repens* infections were identified in Italy, with only four involving the spermatic cord in adults [11]. More recent literature reviews have identified only 20–28 cases of male genital dirofilariasis worldwide, with fewer than 10 occurring in children [7,12]. The reasons for this unusual localization remain speculative; proposed theories include the cooler temperature of the scrotum favoring parasite survival or a potential affinity of the parasite for sex hormones [1]. In our patient, the clinical presentation was misleading, initially suggesting common conditions like orchiepididymitis or testicular torsion, highlighting the diagnostic challenge these cases pose.

The epidemiological context is crucial for diagnosis [13]. The patient had traveled to Sri Lanka for one month, nine months prior to his symptoms. Both Italy and Sri Lanka are highly endemic for *D. repens* [4]. Italy alone accounts for approximately half of all human cases reported in Europe [5], and the parasite has been identified even in small Sicilian islands like Linosa [14]. However, the likelihood of acquiring the infection in Sri Lanka is high. In Sri Lanka, unlike in many other regions, the infection is most common in children under 9 years old, and the male genitalia are frequent sites for the worms [15]. This distinct regional epidemiology, combined with a compatible incubation period of 6–8 months, points toward Sri Lanka as the probable source of infection, though acquisition in Italy cannot be ruled out.

The inflammatory reaction seen in this patient is a characteristic feature of these infections and is often linked to the host’s immune response against a dying or degenerating worm, which can lead to the release of worm antigens or symbiotic Wolbachia bacteria [16]. In our case, we did not identify Wolbachia antigens. Imaging played a key role in guiding the diagnosis. While initial ultrasound findings were non-specific, the follow-up imaging revealed a nodule containing linear hyperechogenic structures. Although worm motility was not observed, this “worm-in-a-bag” sign is highly suggestive of an encysted helminth [17]. Awareness of these sonographic features is critical for clinicians to distinguish these lesions from tumors, as misdiagnosis has previously led to unnecessary orchiectomies [6,18].

Definitive diagnosis of dirofilariasis is typically achieved through surgical excision and histopathological examination of the nodule [1,4]. The pathognomonic histological finding is the presence of the parasite’s thick, multi-layered cuticle [19]. While PCR has been a valuable diagnostic tool since the 1990s [20], some authors have reported discrepancies with histopathology, underscoring the importance of interpreting molecular results in conjunction with clinical and pathological findings [21,22]. In our case, non-specific histology showing helminth fragments prompted the use of PCR, which detected *D. repens* DNA.

Surgical removal is generally considered both diagnostic and curative for human dirofilariasis [4]. In most reported cases, including pediatric ones, no post-excision antiparasitic medications were administered [7,12]. Pharmacological therapy (e.g., albendazole or ivermectin) is typically reserved for cases of incomplete removal or disseminated infection [1,23]. Given the complete excision of the lesion, we did not prescribe further anti-helminthic therapy. The successful outcome in this case was due to a multidisciplinary approach that prevented misdiagnosis and ensured targeted care.

## 4. Conclusions

Our case highlights the importance of including *D. repens* infection in the differential diagnosis of unexplained inguinal masses, particularly in patients from or with a travel history to endemic areas. Although localization in the spermatic cord is rare, it should be considered to prevent misdiagnosis and inappropriate interventions like orchiectomy or prolonged antibiotic courses. A multidisciplinary approach involving surgeons, infectious disease specialists, and pathologists was crucial for the successful management of this case. This case demonstrates that a high index of suspicion based on clinical and epidemiological grounds is essential. In most instances, surgical excision is curative, and further medical therapy is unnecessary.

## Data Availability

Data are contained within the article (and Supplementary Materials).

## Supplementary Materials

The following supporting information can be downloaded at https://www.mdpi.com/article/10.3390/tropicalmed10070184/s1, Table S1: Oligonucleotide primers used in the single-step multiplex PCR targeting the mitochondrial cytochrome c oxidase subunit I (coxI) gene for differential detection of *Dirofilaria immitis*, *D. repens* and *Acanthocheilonema reconditum*. Primer sequences are listed 5′→3′ together with the target parasite, expected amplicon size and primary reference.
