# Supplementary material for: Dirofilaria repens in the Spermatic Cord of a 5-Year-Old Boy: A Rare Pediatric Case in Italy
Source: Trop Med Infect Dis. 2025 Jun 29;10(7):184. doi: 10.3390/tropicalmed10070184 (PMC12299199; doi:10.3390/tropicalmed10070184)
Supplement: Supplementary file 1 [file tropicalmed-10-00184-s001.zip › tropicalmed-3693308-supplementary.pdf]

Target parasite (coxI)	Primer name	Sequence 5′ → 3′	Expected product (bp)	References
Dirofilaria immitis	**Dim-COI-FWD**	**ACCGGTGTTTGGGATTGTTA**	169 bp	(8)
Dirofilaria repens	**Drep-COI-FWD**	**GTATAATTTTGGGTTTACATA CTGTA**	479 bp	(8-9)
Acanthocheilonema reconditum	**Arec-COI-FWD**	**ATCTTTGTTTATGGTGTATC**	589 bp	(8)
(common to all)	**NTR-REV**	**ATAAGTACGAGTATCAATATC**	—	(8)

Table S1 - Oligonucleotide primers used in the single-step multiplex PCR targeting the mitochondrial cytochrome c oxidase subunit I (coxI) gene for differential detection of *Dirofilaria immitis*, *D. repens* and *Acanthocheilonema reconditum*. Primer sequences are listed 5′→3′ together with the target parasite, expected amplicon size and primary reference

- Annealing temperature: 50 °C for all four primer pairs.
- Polymerase & cycling conditions: exactly as in Latrofa et al. (2012).
